# A review focusing on the role of pyroptosis in prostate cancer

**DOI:** 10.1097/MD.0000000000036605

**Published:** 2023-12-15

**Authors:** Zhewen Liu, Shida Kuang, Qihua Chen

**Affiliations:** a Hunan University of Chinese Medicine, Changsha, People’s Republic of China; b The First Affiliated Hospital, Hunan University of Chinese Medicine, Changsha, People’s Republic of China.

**Keywords:** gasdermin, prediction, prostate cancer, pyroptosis, treatment

## Abstract

As one of the types of programmed cell death, pyroptosis has become a focus of research in recent years. Numerous studies have shown that pyroptosis plays a regulatory role in tumor cell invasiveness, differentiation, proliferation, and metastasis. It has been demonstrated that pyroptosis is involved in the regulation of signaling pathways implicated in the pathogenesis of prostate cancer (PCa). Furthermore, the loss of expression of pyroptosis-related genes in PCa has been reported, and pyroptosis-related genes have demonstrated a considerable ability in predicting the prognosis of PCa. Therefore, the potential role of pyroptosis in regulating the development of PCa warrants further investigation and attention. In this review, we summarize the basics of the role of pyroptosis and also discuss research into the mechanisms of action associated with pyroptosis in PCa. It is hoped that by exploring the potential of the pyroptosis pathway in intervening in PCa, it will provide a viable direction for the diversification of PCa treatment.

## 1. Introduction

According to the updated 2022 U.S. cancer statistics, prostate cancer (PCa), the most common type of cancer in the male genitourinary system, is expected to be the number 1 cancer in terms of new cases (27%) and the number 2 cancer in terms of deaths (11%).^[1]^ Nearly 400,000 people die of PCa each year at present and this number is expected to be at least doubled by 2040.^[2]^ The risk of PCa increases progressively with age and more than 85% of new cases originate in patients over 60 years,^[3]^ making it a major source of disease burden for the elderly men.^[4]^ The majority of PCa grow in localized areas and produce symptoms of localized growth, which does not cause death in men.^[5]^ The most common cause of death in patients with PCa is distant metastases of cancer cells, such as metastasis to the spine, bladder, liver, lungs and brain.^[6]^ The key treatment options for PCa include radical prostatectomy, chemotherapy, androgen deprivation and radiotherapy.^[7]^ More specifically, for localized PCa, the primary treatments are active surveillance, radical prostatectomy or ablative radiotherapy, which have been demonstrated to produce significant therapeutic effects.^[2,8]^ The American Cancer Society data shows that the 5-year survival rate is close to 100% in patients with localized PCa, but is only 30% in patients with distant metastases.^[9,10]^ It can be seen that patients suffering from PCa with distant metastases have poor overall survival. The most essential treatment for advanced and metastatic PCa is the androgen deprivation therapy (ADT), as androgens have been shown to help with early PCa progression by stimulating PCa cell proliferation and inhibiting apoptosis.^[11–13]^ ADT is highly effective in the initial stage of treatment, but this approach not only fails to cure PCa, but also often leads to progression to the castration-resistant PCa stage.^[14,15]^ In addition, ADT may cause a variety of adverse effects, such as reduced stamina, increased fatigue, sexual dysfunction, fractures, cardiovascular disease and cognitive impairment, which often lead to poor drug tolerance.^[2,16–20]^ Moreover, PCa patients receiving broad-spectrum chemotherapy or other targeted therapies are prone to multidrug resistance, which accounts for more than 90% of deaths.^[21,22]^ The existence of ADT and multidrug resistance has aroused a search for more alternatives to inhibit cancer cell proliferation and promote cancer cell death.

Pyroptosis was first discovered in 1992 in macrophages infected with the Gram-negative bacterium Salmonella.^[23]^ Cookson et al found that such type of cell death behaves quite differently from apoptosis.^[24]^ In 2001, D’Souza et al used the term “pyroptosis” to describe this pro-inflammatory programmed cell death.^[25]^ The occurrence of pyroptosis is characterized by cell swelling, cell membrane perforation, cell content spilling, chromatin condensation and DNA breakage, and meanwhile, the cell nucleus becomes condensed but remains intact.^[26–28]^ A number of studies have demonstrated that pyroptosis can affect the proliferation, invasion and metastasis of tumor, as well as the tumor microenvironment and immunity, and various anti-cancer drugs based on the pyroptosis pathway are being actively developed.^[29–32]^ Thus, regulating pyroptosis in PCa may be an effective anti-cancer strategy, making it important and valuable to understand the mechanism of pyroptosis and its role in PCa.

In this review, we focus on the mechanisms underlying cellular pyroptosis and explore its role in PCa, with the aim of identifying potential applications for targeting pyroptosis in interventions for this disease. Our hope is that these findings will provide a promising avenue for diversifying treatment options for PCa.

## 2. Molecular mechanism of pyroptosis

**Figure 1.**
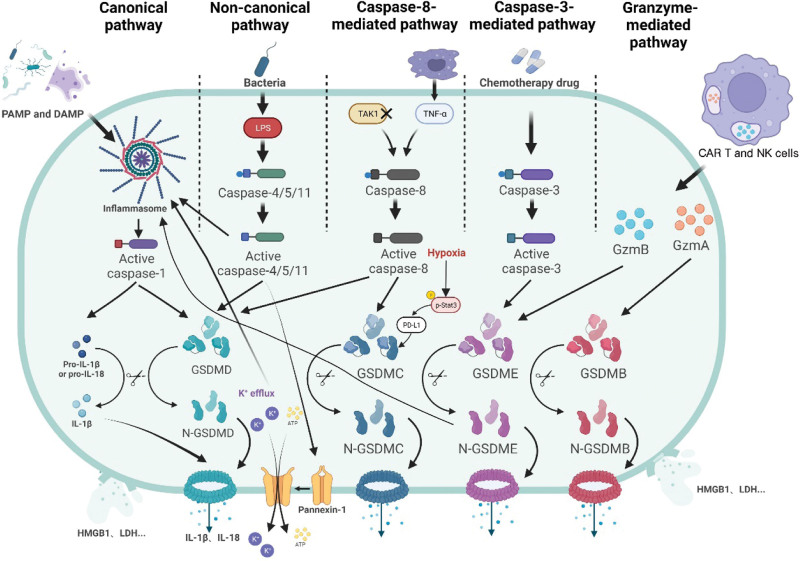
Molecular mechanism of pyroptosis. In the canonical pathway, the PRR recognizes PAMPs and DAMPs and then assembles into inflammasome and leads to caspase-1 activation. Activated caspase-1 further cleaves GSDMD and pro-IL-1β/18. N-GSDMD forms nonselective pores in the cell membrane leading to water influx, IL-1β/18 efflux, cell lysis and death. In addition, cell lysis releases DAMPs, such as HMGB1 and LDH. In the non-canonical pathway, LPS induces caspase-4/5/11 activation, which further leads to pyroptosis by GSDMD cleavage. Furthermore, activated caspase-4/5/11 activates Pannexin-1 and leads to further K + and ATP efflux, which induces the classical pathway of pyroptosis triggered by inflammasome. In addition, activated caspase-11 also activates in inflammasome to promote IL-1β/18 maturation and release. In the caspase-3-mediated pathway, activated caspase-3 induces pyroptosis through cleavage of GSDME, and N-GSDME activates inflammasome to trigger the classical pathway. In the caspase-8-mediated pathway, TNF-α activates caspase-8 to induce pyroptosis, but this requires PD-L1 is transferred to the nucleus and co-regulation of GSDMC transcription with p-Stat3 to occur under hypoxic conditions. In addition, inhibition of TAK1 induces caspase-8 activation, which cleaves GSDMD leading to pyroptosis. In the granzyme-mediated pathway, CAR T and NK cells release GzmB to activate cysteinase-3 in target cells, which then cleaves GSDME to cause pyroptosis. GzmA in cytotoxic lymphocytes can enter target cells to cleave GSDMB and induce pyroptosis. DAMPs = danger-associated molecular patterns, HMGB1 = high mobility group box 1, IL-1β = interleukin-1β, LDH = lactate dehydrogenase, LPS = lipopolysaccharides, PAMPs = pathogen-associated molecular patterns, PD-L1 = programmed death-ligand 1, PRR = pattern recognition receptor, TAK1 = transforming growth factor-β-activated kinase 1, TNF-α = tumor necrosis factor-α.

Pathways related to the mechanisms of pyroptosis can be divided into the canonical pathway, the non-canonical pathway, the caspase-3-mediated pathway, the caspase-8-mediated pathway and the granzyme-mediated pathway (Fig. 1).

### 2.1. Canonical pathway

The canonical pathway of pyroptosis is mediated by the assembly of inflammasomes followed by the cleavage of gasdermin (GSDM) D proteins and the release of interleukin-1β (IL-1β) and interleukin-18 (IL-18).^[33–36]^ Inflammasomes are multi-protein complexes composed of intracytoplasmic pattern recognition receptors (PRRs), effector proteins, and an apoptosis-associated speck-like protein containing the caspase activation and recruitment domain (CARD) (ASC), which is an important component of natural immune cells.^[37]^ There are 8 main types of inflammasome identified, namely nucleotide-binding oligomerization domain-like receptor thermal protein domain associated protein (NLRP)1 inflammasome, NLRP3 inflammasome, NLRP6 inflammasome, NLRP7 inflammasome, NLRP9b inflammasome, NLRC4 inflammasome, pyrin inflammasome and absent in melanoma 2 (AIM2) inflammasome.^[37,38]^ As a family of bio-conserved genes, GSDMs have been found to exert a pore-forming effect, which can lead to membrane permeabilization and lytic pro-inflammatory cell death (i.e., pyroptosis).^[39]^ Six members of the human GSDM family have been identified to date, including GSDM A/B/C/D/E and PJVK.^[40]^ All members of the GSDM family, except pejvakin, consist of an N-terminal cytotoxic domain, a C-terminal inhibitory domain and a flexible linker.^[41,42]^ The N-terminal and C-terminal structural domains interact directly to maintain the GSDM in an inhibited state.^[43]^ The protein at the flexible linker is hydrolytically cleaved by proteases to release an N-terminal structural domain that binds to acidic phospholipids in the cell membrane and forms pores in the membrane containing 16 symmetric protomers, leading to IL-18/1β release and water influx to further cause cell swelling and osmotic lysis, which is known as pyroptosis.^[39,43–45]^ Thus, the formation of pores in the cell membrane by the N-terminal structural domain of GSDM is essential for the occurrence of pyroptosis.^[43]^

In the pyroptosis canonical pathway, the PRR recognizes pathogen-associated molecular patterns and danger-associated molecular patterns first and then recruits ASC to bridge itself to pro-caspase-1, at which point the inflammasome assembly is complete, leading to the self-cleavage activation of caspase-1.^[46,47]^ Some PRRs contain CARD, which can also directly recruit pro-caspase-1 and activate it.^[38]^ Activated caspase-1 is further hydrolyzed into 2 fragments and forms a dimer to become a mature cleaved caspase-1,^[48]^ which cleaves the protein at the ASP275 site of GSDMD to shear the GSDMD into a 22 kDa C-terminus (C-GSDMD) and a 31 kDa N-terminus (N-GSDMD).^[49]^ N-GSDMD binds to the acidic phospholipids of the cell membrane, which in turn forms a gap in the membrane containing 16 symmetric protomers to induce the release of IL-18, IL-1β and water influx, resulting in swelling and pyroptosis.^[39,43–45]^ On the other hand, activated caspase-1 also shears the precursors of IL-1β and IL-18 to become mature IL-1β and IL-18, which are released from the cell through pores formed by GSDMD.^[44,50,51]^ In addition, danger-associated molecular patterns, such as the protein high mobility group box 1 (HMGB1) and lactate dehydrogenase, are released after focal death, leading to increased inflammation and recruitment of immune cells in the tissue.^[52–54]^ Although GSDMD is required for HMGB1 release following inflammatory vesicle activation, the pores in cell membrane formed by GSDMD are not a direct conduit for HMGB1 release, as HMGB1 release occurs only after cell membrane disruption.^[55]^ Interestingly, activation of inflammasomes is found to cause pyroptosis and the release of inflammatory factors, but not necessarily cell death, a specific mechanism that is not yet known.^[32]^

### 2.2. Non-canonical pathway

In the non-canonical pyroptosis pathway, lipopolysaccharides (LPS), either intracellular or from Gram-negative bacteria, bind directly to the CARD structural do-main of human caspase-4/5 and mouse orthologs caspase-11, following which caspases are activated.^[56]^ The activated caspase cleaves the execution protein GSDMD at Asp276 into N-GSDMD, which is then oligomerized and transferred to the cell membrane to eventually form a plasma membrane pore.^[57]^ Notably, the auto-processing of pro-caspase-4/11 to generate P10 product is required for the cleavage of GSDMD and the induction of pyroptosis, which may be a mechanism for caspase-specific recognition of GSDMD.^[58]^ However, activated caspase-4/5/11 cannot directly process IL-1β and IL-18 precursors, but caspase-11 can mediate the maturation and release of IL-1β/IL-18 via activation of NLRP3.^[59]^ In addition, the cleavage of GSDMD by activated caspase-4/5/11 leads to K + efflux, which can induce pyroptosis in the NLRP3 inflammasome assembly via the canonical pathway.^[60–62]^ Moreover, Pannexin-1 has been identified as a key protein in another caspase-11-induced non-canonical pathway of pyroptosis.^[63]^ In this pathway, caspase-11, upon activation by LPS stimulation, can specifically shear and modify Pannexin-1, causing K + efflux and intracellular ATP release, which can further stimulate caspase-1 activation by NLRP3 inflammatory vesicles to induce pyroptosis.^[63]^

### 2.3. Caspase-3-mediated pathway

For many years, activation of caspase-3 has been considered a major feature in the process of apoptosis. However, recent findings have indicated that caspase-3 is actually not specific to apoptosis as activation of caspase-3 can also induce pyroptosis in cells.^[40]^ Previous studies showed that chemotherapeutic drugs could mediate the cleavage of GSDME into N-GSDME and induce pyroptosis via activation of caspase-3.^[64,65]^ In addition, tumor necrosis factor-α (TNF-α) can also activate caspase-3, which then cleaves GSDME-induced pyroptosis at Asp267 or Asp270.^[65]^ Production of N-GSDME by the caspase-3 cleavage of GSDME is found to activate inflammasome to trigger the classical pathway, thereby promoting the maturation and release of IL-1β and IL-18.^[65]^ Besides GSDME, caspase-3 has not been found to mediate the induction of pyroptosis by other GSDM-related molecules for the time being.

### 2.4. Caspase-8-mediated pathway

Caspase-8 was previously thought to be unable to stimulate GSDM to induce pyroptosis. However, in Pathogenic Yersinia-infected mouse macrophages, it was revealed that the effector protein YopJ inhibited the transforming growth factor-β-activated kinase 1 and induced caspase-8 activation, which further cleaved GSDMD to cause cellular scorching, and that the cleaved GSDMD contributed to the NLRP3 inflammatory vesicle-dependent IL-1β release.^[66,67]^ In addition, caspase-8 was found to be recruited to the Naip5/NLRC4/ASC inflammasome when mouse macrophages were exposed to Legionella pneumophila, but it was only activated upon caspase-1 or GSDMD inhibition and its activation would trigger the GSDMD-independent pore formation by pyroptosis.^[68]^ Moreover, it is worth noting that programmed death-ligand 1 (PD-L1) can convert TNF-mediated apoptosis into pyroptosis in breast cancer cells.^[69]^ Under hypoxic conditions, p-Stat3 promotes the nuclear translocation of PD-L1 to further enhance GSDMC transcription, at which point TNF-α stimulates caspase-8 to specifically cleave GSDMC to produce N-GSDMC and form pores in the cell membrane, thereby inducing pyroptosis.^[69]^ Obviously, the TNF-α-induced tumor pyroptosis requires the simultaneous presence of nuclear PD-L1, caspase-8 and GSDMC.

### 2.5. Granzyme-mediated pathway

Granzymes are serine proteases in the cytoplasmic granules released by cytotoxic lymphocytes and natural killer cells. Recent studies have shown that granzyme B can directly cleave GSDME to release the cytotoxic N-terminal structural domain N-GSDME, thereby contributing to cell scorching.^[70]^ Granzyme B from chimeric antigen receptor T cells has been found to activate caspase-3 in target cells, which mediates pyroptosis through cleavage of GSDME.^[71]^ Lymphocyte-derived granzyme A is also capable of cleaving GSDMB to produce pore-forming N-GSDMB in GSDMB-expressed cancer cells, so as to induce pyroptosis in cancer cells.^[72]^ The granzyme-mediated pyroptosis in cancer cells may amplify inflammatory signals in the tumor microenvironment and promote the recruitment of immune cells to stimulate anti-tumor immunity, which may be a new direction for the future development of tumor therapy.^[30]^

## 3. Role of pyroptosis in PCa

### 3.1. Pyroptosis involvement in PCa development

**Figure 2.**
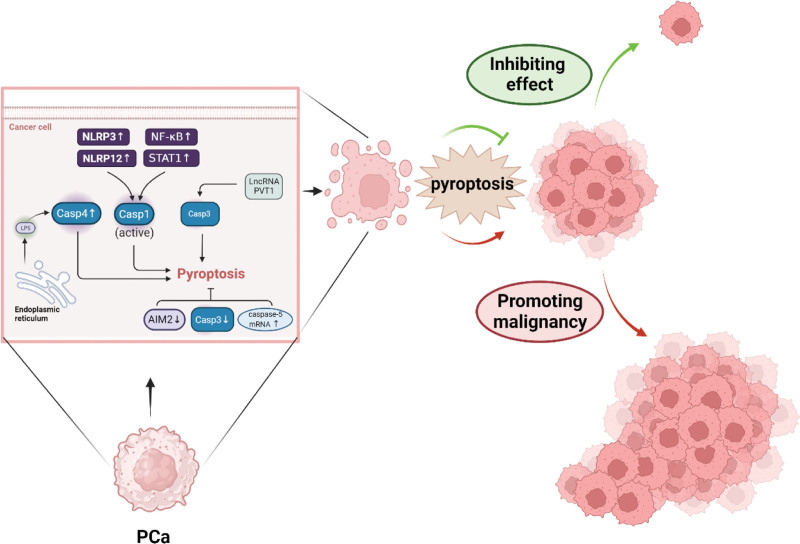
Pyroptosis involvement in prostate cancer development. Not all pyroptosis-related pathways are activated in PCa, and some of them are actually inhibited. The occurrence of pyroptosis in PCa cells may play a dual role in the progression of PCa. AIM2 = absent in melanoma 2, Casp = caspase, LSP = lipopolysaccharides, NF-κB = nuclear factor-κ-gene binding, NLRP = NOD-like receptor thermal protein domain associated protein, PCa = prostate cancer, PVT1 = plasmacytoma variant translocation 1, STAT1 = signal transducers and activators of transcription 1.

The development of PCa is accompanied by a range of cellular processes, including cell proliferation, migration, and apoptosis.^[5]^ Pyroptosis has emerged as a prominent research topic in recent years, with multiple studies demonstrating its pivotal role in modulating signaling pathways implicated in the development of PCa.^[73]^ The major pyroptosis pathways implicated in PCa encompass the caspase-1, caspase-3 and caspase-4/5/11 signaling cascades.^[65,74]^ Xu et al observed an upregulation of NLRP3 expression in both PCa tissues and cell lines, as well as a promotion of PCa malignancy through NLRP3 overexpression. Conversely, inhibition of NLRP3 via Caspase-1 led to a suppression of malignant progression in PCa cell lines.^[75]^ Activation of NLRP12 in PCa regulates Caspase-1, leading to the activation and secretion of IL-1β and IL-18. These cytokines promote the development of PCa by enhancing inflammation and immune evasion.^[76]^ Liu et al discovered that nuclear factor-κ-gene binding and signal transducers and activators of transcription 1 can induce pyroptosis in PCa cells by regulating the expression of caspase-1 or GSDMD proteins, thereby releasing a large amount of inflammatory factors to suppress tumor growth.^[77]^ A significantly elevated level of caspase-5 mRNA expression was observed in PCa patients as compared to normal subjects.^[78]^ Another study found a significant reduction in the expression of caspase-3 in PCa tissue.^[79]^ Long non-coding RNA (LncRNA) plasmacytoma variant translocation 1 has been implicated in the progression of PCa, and suppression of plasmacytoma variant translocation 1 expression significantly enhances Caspase-3 expression in PCa tissues.^[80]^ The AIM2 inflammasome is an innate immune signaling complex that activates the classical pathway of pyroptosis. Notably, AIM2 expression levels are significantly reduced in PCa and even undetectable in some PCa cell lines.^[81]^ In addition, it has been discovered that alterations in the structure of the endoplasmic reticulum in PCa can stimulate LPS synthesis, leading to increased expression of Caspase-4, inducing pyroptosis and inhibiting tumor growth.^[82]^ It can be observed that not all pyroptosis-related pathways are activated in PCa, and some of them are actually inhibited. The occurrence of pyroptosis in PCa cells may play a dual role in the progression of PCa (Fig. 2). The discussion should be based on the characteristics of cancer development in different stages. Based on current evidence, pyroptosis exerts a greater promotional effect rather than inhibitory effect during the PCa stage.

### 3.2. Non-coding RNAs (ncRNAs) in PCa pyroptosis

**Figure 3.**
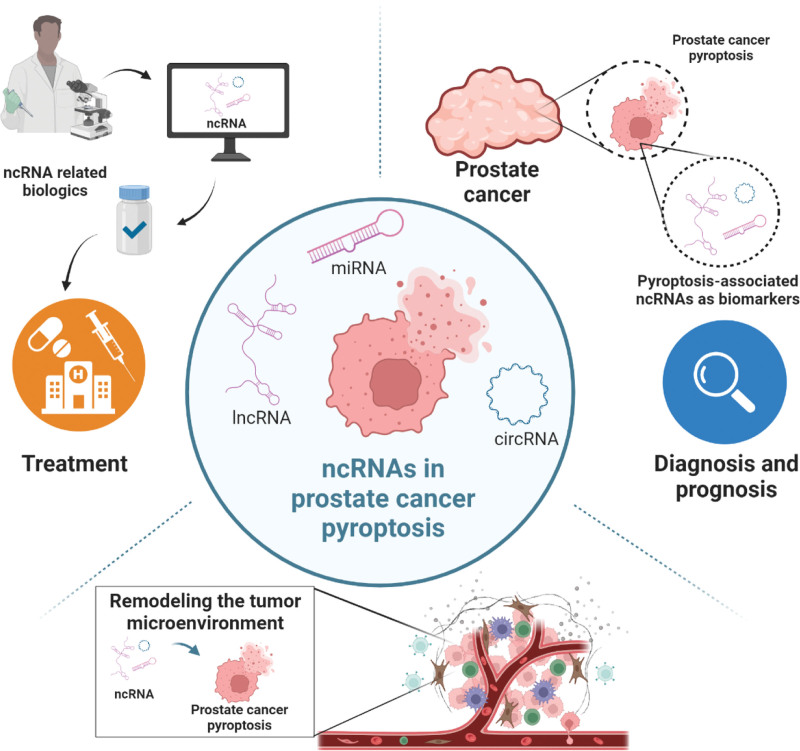
Non-coding RNAs in prostate cancer pyroptosis. NcRNAs are intimately linked with pyroptosis in prostate cancer, and these pyroptosis-associated ncRNAs have the potential to remodel the tumor microenvironment and serve as valuable tools for tumor diagnosis and treatment. ncRNAs = non-coding RNAs.

The ncRNAs are single-stranded nucleic acid molecules that do not code for proteins and mainly include microRNAs, lncRNAs, and circular RNAs. These molecules serve as key regulators of physiological and pathological processes in organisms.^[83–85]^ A large number of studies have shown that ncRNAs are particularly relevant to cancer and have been identified as oncogenic drivers and tumor suppressors in certain cancers, demonstrating great potential in the prognosis, diagnosis, and treatment of tumors.^[86–88]^ The ncRNAs are also involved in the pyroptosis of tumor cells. NcRNAs also play a crucial role in the process of tumor cell pyroptosis, contributing to the remodeling of the tumor microenvironment by inducing chronic inflammation and promoting anti-tumor immunity.^[89]^ Tissue specificity is a key feature of ncRNAs, and dysregulation of their expression has been observed in various types of tumors. This enables the use of ncRNAs as biomarkers for tumor diagnosis and prognosis. PCa3 is the first and only ncRNA to have received approval from the U.S. Food and Drug Administration for testing as a cancer biomarker.^[88]^ The development of tumor drug resistance currently significantly limits the therapeutic efficiency of current anti-tumor drugs. Therefore, the development of effective therapies for oncogenes or ncRNAs used as tumor suppressors has been an active area of research in recent years.^[90,91]^ It has been suggested that lncRNAs play a special role in the development of PCa and the progression of endocrine therapy resistance, and will be a promising therapeutic target for advanced PCa.^[92]^ A recent study established a model for efficient prediction of PCa based on pyroptosis-associated genes and found that the pyroptosis-associated gene LncRNA AC005253.1 was highly correlated with PCa. In vitro experiments revealed that silencing AC005253.1 inhibited the proliferation, migration, and invasion of PCa cells.^[93]^ It is evident that ncRNAs associated with scotomies can offer new avenues for clinicians to develop novel drugs. Overall, the role of ncRNAs in the process of cancer cell pyroptosis is intricate, and further experiments are required to investigate precise interventions in different ncRNA-mediated cell pyroptosis and develop more biomarkers and therapeutic targets (Fig. 3).

### 3.3. Interventional pyroptosis regulates PCa

In recent years, extensive studies have reported the ability of a variety of drugs to induce pyroptosis in cancer cells and the evidence that the occurrence of pyroptosis can inhibit tumor growth.^[94]^ For example, the decitabine treatment is capable of enhancing chemosensitivity in breast cancer by increasing the level of GSDME expression in tumor cells.^[95]^ Similarly, Wang et al treated NCI-H522 lung cancer cells with chemotherapeutic agents including adriamycin, actinomycin-D, bleomycin and topotecan and found that the GSDME-dependent pyroptosis occurred in cancer cells upon activation of caspase-3 by chemotherapy drugs.^[65]^ Notably, the results of a controlled pyroptosis induction experiment via the nanoparticle-mediated selective delivery of active GSDM protein to cancer cells showed that pyroptosis cell death in only 10% of tumor cells would be sufficient for clearing the entire tumor graft.^[96]^ Caspase-3 can be activated by chemotherapy or targeted drugs in PCa tissue to cleave GSDME, thereby inducing pyroptosis to provide anti-tumor effects.^[97]^ In addition, drugs that target and activate NLRP3 have also been found to cause PCa pyroptosis by modulating caspase-1 and its downstream IL-1β and IL-18, which then limits the extent and scope of inflammation.^[76]^ Zhang et al designed and synthesized a novel 3′,5′-diprenylated chalcone, which was demonstrated to exert proliferation-inhibiting and apoptosis-promoting effects on PCa cells. The authors further found that this substance successfully triggered the GSDME-dependent pyroptosis by inhibiting the protein kinase C δ/c-Jun N-terminal kinase pathway to produce anti-PCa effects.^[98]^ Yu et al identified AC005253.1, an LncRNA associated with PCa pyroptosis, and reported that the inhibition of AC005253.1 expression suppressed the viability, migration and invasion of PCa cells, significantly increased the expression of AIM2 inflammasome, and promoted the pyroptosis in PCa cells.^[93]^ In view of the findings above, the induction of pyroptosis in PCa cells can be targeted as a new strategy to inhibit tumor growth.

### 3.4. Predictive markers for pyroptosis-related PCa

With the improvement of public awareness on prostate-specific antigen (PSA) screening and physical examinations, an increasing number of patients have received radical local treatment, but 30% to 40% of the PSA patients still experienced biochemical recurrence after local treatment or metastasis.^[99,100]^ Early detection of biochemical recurrence is important for the management and treatment of patients with PCa. Although numerous predictors of prognosis for PCa patients, such as the Gleason score and PSA, have been established, they have limited ability in predicting the time to biochemical recurrence.^[101]^ Therefore, developing a highly accurate and specific biomarker is essential for predicting PCa prognosis and guiding its treatment. Currently, studies based on pyroptosis-related genes (PRGs) in the prognosis of PCa have been showing encouraging application outcomes. Many researchers have used bioinformatics to investigate the potential value of PRGs in predicting prognosis in PCa patients (Table 1). For example, Hu et al established a risk signature based on PRG clusters containing 8 genes using the expression profile and clinical data. With the nomogram scoring system, the likelihood of progression-free survival at 1, 3, and 5 years could be predicted.^[107]^ Similarly, Wang et al constructed and validated a risk model based on 8 PRGs, which demonstrated that pyroptosis was associated with tumor immune cell infiltration in PCa.^[104]^ Zhang et al established a signature consisting of 7 genes by Lasso regression analysis. It can effectively stratify the risk of PCa patients into subgroups that are more suitable for immunotherapy and potentially serve as indicators of response to immunotherapy for PCa.^[106]^ Li et al, by building a prognostic model with 5 PRGs, validated that the risk scores of these 5 PRGs were independent factors for relapse in PCa patients and were found to be related to the immune microenvironment.^[105]^ Fu et al constructed 2 different pyroptosis patterns based on extensive RNA-seq and scRNA-seq data from PCa patients. The authors established a novel prognostic gene signature for predicting the recurrence free survival in PCa patients, which was shown to be an independent factor associated with the recurrence free survival in PCa.^[103]^ Xiao et al established a prognostic risk model using a 6-hub PRG. By analyzing its relationship with PCa in terms of clinicopathological characteristics, tumor immune microenvironment and response to drug treatment, it was found that this risk model was of good clinical significance.^[102]^ Luo et al constructed a risk prediction model containing 6 PRGs based on a relevant database, and reliability assessment suggested that ZDHHC1 accounted for considerable weighting. Further research of the anti-tumor effects of ZDHHC1 confirmed its inhibitory effects on the migration, invasion and proliferation of PCa cells.^[108]^ In addition, the long-stranded ncRNAs associated with pyroptosis are also found prognostic. Yu et al established a prognostic model based on 6 long-stranded ncRNAs associated with pyroptosis and determined their expression levels in PCa cells. Further experimental validation showed that LncRNA AC005253.1 could affect the proliferation, migration and invasion abilities of PCa cells. Besides, AC005253.1 might play an important role in PCa by affecting pyroptosis through the AIM2 inflammasome.^[93]^ The absence of comprehensive and individualized treatment options contributes significantly to the elevated mortality rates observed in patients with metastatic PCa. The characterization of pyroptosis as a specific cell death pathway and its related gene expression has shown great potential for predicting the risk of PCa. Pyroptosis-associated genes could potentially serve as prognostic markers for PCa in the future.

**Table 1:** PRGs in the prognosis of prostate cancer.

Signature	Data source	Validation data	Experimental verification	References
CHMP4C ↑, GSDMB ↑, NOD2 ↑, PLCG1 ↑, CYCS ↑ and GPX4 ↑	TCGA, cBioportal and MSigDB	TCGA cohort and GSE21032	Yes	^[102]^
AC129507.1 ↓, AC005253.1 ↑, AC127502.2 ↑, AC068580.3 ↑, LIMD1-AS1 ↑ and LINC01852 ↓	UCSC Xena, GEO and TCGA	GSE116918	Yes	^[93]^
CASP8 ↑, GSDMB ↑, BAK1 ↑, BAX ↑, CHMP4B ↓, CHMP4C ↑, CHMP6 ↓, TP53 ↑, TP63 ↓, CASP9 ↑, GPX4 ↑ and PLCG1 ↑	UCSC Xena, GEO, ICGC and MSigDB	TCGA-PRAD cohort and ICGC-PRAD cohort	Yes	^[103]^
PTGS2 ↑, CLDN1 ↑, 和 RPPH1 ↑, IL7R ↓, COL22A1 ↓, ZNF185 ↓, SRD5A2 ↓ and AOX1 ↓	TCGA and GEO, USUC Xena and MSigDB	Real samples	Yes	^[104]^
BAK ↑, BAX ↑, CHMP7 ↑, GSDMB ↓ and NLRP1 ↓	TCGA and GEO	GSE40272	Yes	^[105]^
UBE2C ↑, KIFC2 ↑, MAPK8IP3 ↑, TTLL3 ↑, MYBL2 ↑, MMP11 ↑ and UBAP1L ↑	TCGA and GEO	GSE21034	Yes	^[106]^
CENPA ↑, LCN2 ↓, COL7A1 ↑, ALB ↑, UBXN10 ↓, SPZ1 ↑, SCNN1A ↓ and TFF3 ↓	TCGA, GEO and MSigDB	GSE70769	N/A	^[107]^
ATG7 ↑, HDAC6 ↑, IRF3 ↑, CHMP1A ↓, IRF1 ↓ and ZDHHC1 ↓	TCGA, GEO, MSigDB, GeneCards and Reactome	GSE54460	Yes	^[108]^

↑ = up-regulation of gene expression. ↓ = down-regulation of gene expression.

GEO = Gene Expression Omnibus, ICGC = international cancer genome consortium, MSigDB = Molecular Signatures Database, NLRP = NOD-like receptor thermal protein domain associated protein, NOD = nucleotide-binding oligomerization domain, PRGs = pyroptosis-related genes, TCGA = The Cancer Genome Atlas, UCSC Xena, University of California Santa Cruz Xena.

## 4. Conclusions and prospects

Recently, pyroptosis, which represents a novel and unique form of cell death, has drawn a great deal of attention for its recognized benefits in anti-cancer treatment. Pyroptosis is mainly characterized by cell swelling, cell membrane rupture and the release of inflammatory cytokines. The process of pyroptosis activation involves the release of cell contents that are toxic to neighboring healthy cells and induction of an inflammatory response, which will ultimately lead to cell death. Pyroptosis is like a mixed blessing in cancer. On the one hand, it facilitates the provision of a nutritious microenvironment for cancer cells, thereby accelerating their growth, but on the other hand, it inhibits tumorigenesis and progression.^[33]^ The dual mechanisms by which pyroptosis-related factors promote and inhibit tumor development remain to be explored.

In this review, we summarize the basics of the role of pyroptosis and also discuss research into the mechanisms of action associated with pyroptosis in PCa. Admittedly, our current understanding of pyroptosis in PCa is only the tip of the iceberg. Research on pyroptosis-based treatment of PCa is still in its infancy and a number of questions are yet unaddressed. Further research should focus on determining whether there are interactions between pyroptosis and other forms of programmed cell death, and the impact of their interaction on PCa treatment. With continuous accumulation of evidence on the role of pyroptosis in PCa, the application of drugs targeting pyroptosis in the treatment of PCa are expected to become more promising. Overall, a more comprehensive mechanistic insight into pyroptosis in the tumor microenvironment will facilitate the development of novel and effective therapies to treat PCa.

## Author contributions

**Conceptualization:** Zhewen Liu, Shida Kuang, Qihua Chen.

**Funding acquisition:** Qihua Chen.

**Project administration:** Qihua Chen.

**Resources:** Zhewen Liu.

**Software:** Shida Kuang.

**Supervision:** Qihua Chen.

**Visualization:** Shida Kuang.

**Writing – original draft:** Zhewen Liu.

**Writing – review & editing:** Zhewen Liu, Qihua Chen.
